# Analysis of changes in microbiome compositions related to the prognosis of colorectal cancer patients based on tissue-derived 16S rRNA sequences

**DOI:** 10.1186/s12967-021-03154-0

**Published:** 2021-11-29

**Authors:** Sukjung Choi, Jongsuk Chung, Mi-La Cho, Donghyun Park, Sun Shim Choi

**Affiliations:** 1 Division of Biomedical Convergence, College of Biomedical Science, Institute of Bioscience & Biotechnology, Kangwon National University, Chuncheon, 24341 Republic of Korea; 2 GENINUS Inc., Seoul, 05836 Republic of Korea; 3 Department of Medical Life Science, College of Medicine, Catholic University of Korea, Seoul, 06591 Republic of Korea

**Keywords:** Colorectal cancer, Microbiome, 16S rRNA, *F. nucleatum*, *B. fragilis*

## Abstract

**Background:**

Comparing the microbiome compositions obtained under different physiological conditions has frequently been attempted in recent years to understand the functional influence of microbiomes in the occurrence of various human diseases.

**Methods:**

In the present work, we analyzed 102 microbiome datasets containing tumor- and normal tissue-derived microbiomes obtained from a total of 51 Korean colorectal cancer (CRC) patients using 16S rRNA amplicon sequencing. Two types of comparisons were used: ‘normal versus (*vs.*) tumor’ comparison and ‘recurrent *vs.* nonrecurrent’ comparison, for which the prognosis of patients was retrospectively determined.

**Results:**

As a result, we observed that in the ‘normal *vs.* tumor’ comparison, three phyla, Firmicutes, Actinobacteria, and Bacteroidetes, were more abundant in normal tissues, whereas some pathogenic bacteria, including *Fusobacterium nucleatum* and *Bacteroides fragilis*, were more abundant in tumor tissues. We also found that bacteria with metabolic pathways related to the production of bacterial motility proteins or bile acid secretion were more enriched in tumor tissues. In addition, the amount of these two pathogenic bacteria was positively correlated with the expression levels of host genes involved in the cell cycle and cell proliferation, confirming the association of microbiomes with tumorigenic pathway genes in the host. Surprisingly, in the ‘recurrent *vs.* nonrecurrent’ comparison, we observed that these two pathogenic bacteria were more abundant in the patients without recurrence than in the patients with recurrence. The same conclusion was drawn in the analysis of both normal and tumor-derived microbiomes.

**Conclusions:**

Taken together, it seems that understanding the composition of tissue microbiomes is useful for predicting the prognosis of CRC patients.

**Supplementary Information:**

The online version contains supplementary material available at 10.1186/s12967-021-03154-0.

## Introduction

Colorectal cancer (CRC), like many other cancers, is a malignant disease that occurs as a result of the accumulation of complex genetic and epigenetic changes. Although it has been reported that the majority of CRC cases (~ 80%) are due to nongenetic or epigenetic changes and less than 20% of CRC cases are caused by genetic mutations [1], these two types of risks are actually entangled in a very complicated way that makes it almost impossible to differentiate between the upstream driver risk and the downstream passenger risk in causing CRC. Genetically, alterations in *Wnt* signaling pathways initiated by *APC* mutation are known as one of the common causes of familial types of CRC [2, 3]. On the other hand, smoking cigarettes, diets rich in red meats and processed foods, and drinking alcohol have frequently been linked to nongenetic environmental risk factors for CRC [4, 5], and the microbiota has recently been added to that list. The microbiome has been proven in several studies to be a mediator between genetic mutations and harmful diets in the onset and progression of CRC.

It is known that abnormal changes in the composition of the gut microbiome can lead to disruption of epithelial barrier function, which increases inflammation and in turn leads to various gastrointestinal diseases, including CRC [6, 7]. In fact, it has been reported that the distribution of the microbiome differs significantly between normal and tumor tissues or between normal and cancer fecal samples, mainly due to dysbiosis of the microbiome under tumorigenic conditions. For instance, pathogenic bacteria such as *Bacteroides (B.) fragilis* and *Fusobacterium (F.) nucleatum* were significantly more enriched in the tumor tissue than in the normal tissue, and conversely, nonpathogenic members of the Bacteroidetes and Firmicutes phyla were more abundant under normal conditions than tumorigenic conditions for both tissue samples and fecal samples [8, 9].

Pathogenic bacteria are known to directly or indirectly cause enhanced inflammation and oxidative DNA damage and even stimulate cancer-causing signaling pathways inside the cell [10–13]. Particularly, according to Strauss et al. [13], Fusobacteria can invade colonic epithelial cells, destroying the epithelial barrier that allows CRC cells to survive or be maintained. In addition, some studies have shown that *F. nucleatum* can activate *Wnt/β*-catenin signaling, promoting cell proliferation and inflammation, through binding of its *FadA* adhesion protein to E-cadherin on the surface of colon cells [14–16], or through activating *TLR4* signaling to *NF-kB* [17]. Likewise*,* another pathogenic bacterium abundant in CRC, *B. fragilis*, also known as an enterotoxin-producing bacterium, can take part in multistep tumorigenesis by producing toxins. Toxins are known to induce E-cadherin degradation, causing downstream β-catenin signaling, and to stimulate the release of reactive oxygen species and the expression of inflammatory cytokines that cause DNA damage [18–20].

Tjalsma et al. [21] proposed a ‘driver-passenger’ model to explain how the microbiome can facilitate CRC tumorigenesis. According to the model, driver pathogenic bacteria induce DNA damage in colon epithelial cells, leading to the initiation of tumorigenesis. Damaged epithelial cells in turn change the surrounding tumor microenvironment such that opportunistic bacteria (i.e., passenger bacteria) with a competitive advantage in this altered tumor microenvironment defeat and replace healthy gut bacteria, eventually worsening inflammation and accelerating cell proliferation, promoting tumorigenesis. It is, however, worth noting that thus far, no single bacterial species has universally been associated with all CRC patients because substantial variations are present in the compositions of microbiota associated with CRC [22, 23]. It seems that changes in both pathogenic and nonpathogenic microbiomes are responsible for the initiation and/or progression of CRC.

In the present work, using the microbiome information estimated from 16S rRNA amplicon sequencing data generated from matched samples of CRC patients, including tumors and adjacent normal tissues derived from the same patient, we investigated compositional changes in microbiomes related to the tumorigenesis of CRC. We also investigated the compositions of microbiomes between nonrecurrent CRC (named ‘crc_nRC’) and recurrent CRC (named ‘crc_RC’), revealing the bacterial population associated with the relapse of CRC.

## Materials and methods

### Sample collection and generation of 16S rRNA sequences

A total of 51 matched normal and tumor samples obtained from the same individuals with CRC (mostly at TNM stage 2 and 3, see Additional file 1: Table S1, aged 43–86, 51 males collected from the cecum to the rectum at the Samsung Medical Center in Seoul, Republic of Korea) who underwent resection surgery were used for producing the host RNA-seq data and 16S rRNA data to investigate gene expression patterns and the composition of microbiomes that the CRC tissues carry. The RNA-seq data and the V3–V4 amplicon sequencing data of 16S rRNAs were obtained with an Illumina MiSeq reagent kit v3 (2 × 300 bp, Illumina, USA). The PCR primers, i.e., forward (CCTACGGGNGGCWGCAG) and reverse (GACTACHVGGGTATCTAATCC), were designed from the hypervariable regions (V3–V4) of 16S rRNAs. PCR was conducted using 2× KAPA HiFi HotStart ReadyMix (Roche) under the following conditions: 95 °C solution chain for 3 min, 25 cycles of 95 °C for 30 s, 55 °C for 30 s, and 72 °C for 45 s, followed by a 72 °C extension for 5 min. Sequencing libraries were then constructed using a TruSeq^®^ DNA PCR-Free Sample Preparation Kit (Illumina, USA) and TruSeq^®^ Nextera XT index primer (Illumina, USA), and 2× KAPA HiFi HotStart ReadyMix (Roche) using the PCR products after purification. Subsequently, paired-end reads were generated by sequencing on the MiSeq platform after determining the quality of the library with the Tapestation 4200 platform (Agilent Technologies) and a Qubit Fluorometer (Thermo Fisher Scientific).

### Analysis of the microbiomes using bioinformatics tools

The sequencing reads were selected by filtering out low-quality sequences, including primer sequences, truncated sequences, and sequences that were classified into Eukarya and Archaea lineages, following a previously reported QIIME (v1.9.1) quality control process [24]. After finishing quality control procedures, an average of 188,342 high-quality reads per sample (median 190,643; range 128,130–256,314) were obtained, where the average length and quality score were 268.1 bp and 33.01, respectively. Then, the paired-end reads were assembled using the Fast Length Adjustment of SHort reads (FLASH) [25] tool, and chimeric sequences were also excluded by matching the clean tag sequences to the reference database using the Usearch software v6.1 algorithm [26]. Eventually, an average of 139,572 clean reads per sample (range 93,441–208,822) were obtained after filtering chimeric reads.

All the cleaned sequences were used for clustering analysis that led to the identification of operational taxonomic units (OTUs) after removing singleton OTUs. The taxonomic rank (i.e., phylum, class, order, family, genus, and species) of each sample was determined using the Ribosomal Database Project (RDP) classifier [27] by aligning the sequence to the GreenGene reference database (release 13.8) [28] at a 97% minimum similarity level. The final OTU table was used to generate a taxonomic profile graph by including only taxa with at least 0.1% relative abundance in each group. See Additional file 2: Table S2: the OTU table used in this study. The compositional characteristics of the microbiomes differentially enriched in normal and tumor tissues were investigated by linear discriminant analysis effect size (LEfSe) [29].

### Estimation of α- and β-diversity

The α-diversity was evaluated by the Shannon index and observed OTUs with QIIME software, while β-diversity was estimated by principal coordinate analysis (PCoA) based on the Bray–Curtis distance [30]. Permutational multivariate analysis of variance (PERMANOVA) as implemented by the ‘*adonis*’ function in the R package ‘Vegan*’* was applied to test the microbial composition between groups. The box plots and diagrams for these analyses were constructed with the ‘ggplot2’ package in R (v3.6.2). All statistical significance tests were performed with the ‘Wilcoxon rank-sum’ test using the R package.

### Prediction of metabolic pathways based on the composition of microbiomes

To predict the functions of bacteria, software called ‘PICRUSt’, i.e., an acronym for ‘phylogenetic investigation of communities by reconstructing of unobserved states’, was used, the main procedures for which were well described previously [31]. The metabolic functions were estimated by mapping the composition of the identified bacteria into the KEGG database. Statistical Analysis for Metagenomic Profiles (STAMP) [32] was used to identify different metabolic functional abundances between groups. A corrected *P-*value < 0.05 was considered to be significant.

### Estimation of differentially expressed genes

After the quality of sequencing reads was determined by FastQC (https://www.bioinformatics.babraham.ac.uk/projects/fastqc/), the low-quality (Phred score < 33) and adaptor sequences were removed by Trimmomatic (v0.39) [33]. The reference genome (GRCh38/hg38) was then indexed by STAR (v2.7.6a) [34]. Subsequently, the cleaned reads were mapped to the indexed reference genome using STAR, following previously reported procedures [35, 36]. The count value for each gene was then estimated using ‘htseq-count’ [37] after gene names were assigned for the mapped reads by the ‘GTF’ file of the ‘GENCODE Gene Set’ (release 30) (https://www.gencodegenes.org/human/release_30.html). Finally, differentially expressed genes (DEGs) estimated by comparing gene expression between normal and tumor conditions were identified using ‘DESeq2’ [38] after the read counts were normalized. Two thresholds, an adjusted *P-*value (i.e., Q-value) < 0.01 and |log2fold change (fc)|> 1 (i.e., abs(log2fc)), were applied to estimate DEGs by comparing gene expression levels between tumors and normal tissues. Principal component analysis (PCA) revealed that tumor and normal samples were clearly distinguished. However, one nonrecurrent sample (10003704) was revealed to be an outlier and was removed from later analysis.

### Gene set enrichment analysis and cellular heterogeneity of host genes

Two annotation methods were used for the analysis of DEGs. (i) The single-sample GSEA (ssGSEA) method, an extension of gene set enrichment analysis (GSEA), was used to calculate separate enrichment scores for each pairing of a sample and gene set. (ii) A cell type deconvolution tool, xCell, was used to analyze cellular heterogeneity between tumors and normal tissues. Subsequently, the specific gene set enrichment score and deconvoluted cell composition information were used for correlation analyses with microbial compositions.

## Results

### Tumors had a lower bacterial diversity than normal tissues

**Fig. 1:**
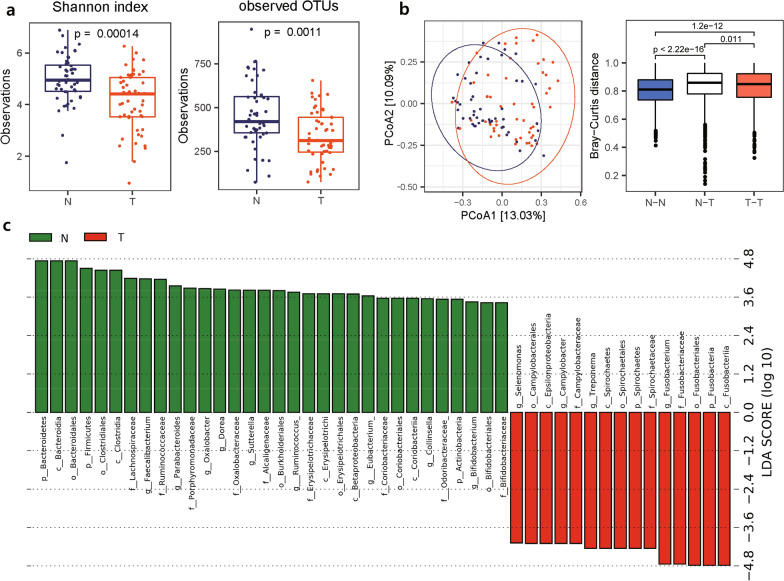
Bacterial diversity of normal and tumor tissues in CRC patients**. a** The α-diversity estimated by the Shannon index and observed OTUs. **b** (left) The β-diversity estimated using PCoA of OTUs. (right) Distribution of Bray–Curtis distances of OTUs in normal samples (N–N), tumor samples (T-T), and normal and tumor samples (N-T). **c** LEfSe plot illustrating microbial taxa enriched in normal compared with CRC tumor tissues. N: normal, T: tumor

From the 16S rRNA amplicon sequencing data obtained from a total of 51 CRC patients with matched normal and tumor tissues, we attempted to identify microbial communities and to estimate their diversity and abundance. We found that α-diversity in tumor tissues was significantly lower than that in normal tissues (Fig. 1a), indicating that the number of inhabiting bacterial species is significantly reduced in the location where tumor formation and progression occur. A possible explanation for this observation is that the tissue environment affected by dysbiosis of the microbiota can be detrimental to some healthy bacteria. The β-diversity estimated using PCoA plots also indicated that the bacterial population structure in tumor tissues was distinct from that in normal tissues, with a significant Bray–Curtis distance (R^2^ = 0.039, *p* = 0.001) (Fig. 1b). Relatedly, using unsupervised hierarchical clustering, we examined whether the population structure of the microbiome was similar between normal tissues and tumors in the same patient or between normal tissues or tumors in different patients (Additional file 1: Fig. S1). Although the clustering pattern was complex in that the clusters in the dendrogram were mixed patterns supporting the former or the latter scenario, the similarity of microbial population structures seemed to be higher between normal or tumor tissues in different patients than between normal and tumor tissues in the same patient.

Subsequently, LEfSe, i.e., linear discriminant analysis (LDA) effect size, was performed to investigate differentially abundant microbiome features (clades, OTUs, etc.) in normal and tumor tissues, and it was confirmed that normal-enriched microbiome features were distinct from tumor-enriched microbiome features (Fig. 1c, Additional file 1: Fig. S2). Namely, in the differential microbiome features ranked by effect size, four genera, *Fusobacterium* (g_Fusobacterium), *Treponema* (g_Treponema)*, Selenomonas* (g_Selenomonas), and *Campylobacter* (g_Camplylobacter), were enriched in tumor tissues, whereas three phyla, Bacteroidetes (p_Bacteroidetes), Actinobacteria (p_Actinobacteria) and Firmicutes (p_Firmicutes) (including Clostridia at its lower class level (c_Clostridia)), were abundant in normal tissues (Fig. 1c). Similarly, hierarchical clustering analysis of bacterial proportions accompanied by a heatmap confirmed that pathogenic bacteria and healthy bacteria were separately grouped into subclusters, indicating that bacteria with similar characteristics coevolved and cooccurred under the influence of an altered environment (Additional file 1: Fig. S3). It is notable that LEfSe-based analysis also adheres to the idea that tumor tissues harbor fewer bacterial features than normal tissues.

### Identification of pathogenic bacteria associated with tumor progression

**Fig. 2:**
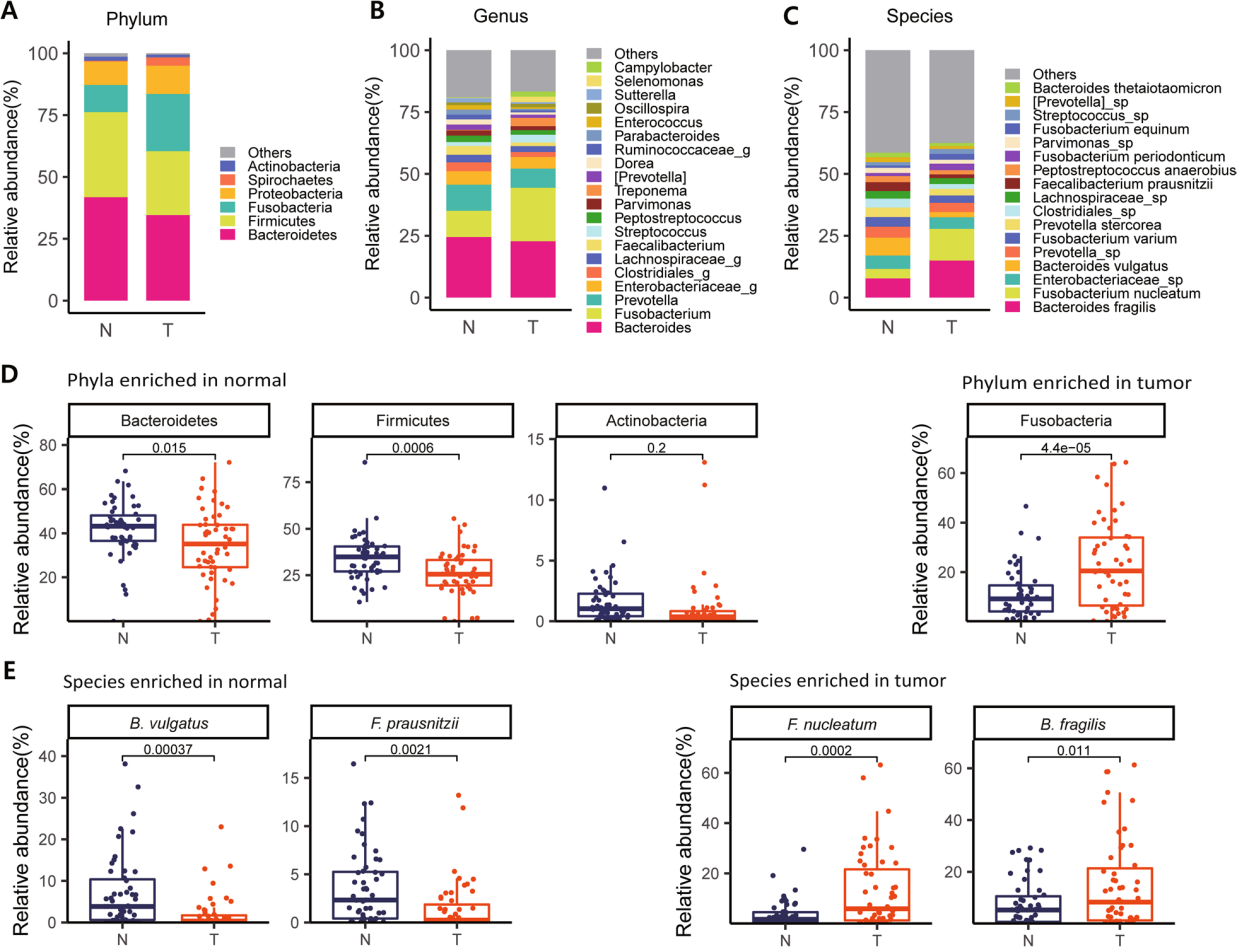
Colorectal cancer-associated bacterial composition. Average relative composition of the bacterial community at the phylum (**a**), genus (**b**) and species levels (**c**). **d** Box plot analysis of the relative abundance of four bacterial phyla, Bacteroidetes, Firmicutes, Actinobacteria and Fusobacteria. **e** Box plot analysis of the relative abundance of four species, *B. vulgatus* and *F. prausnitzii*, *F. nucleatum* and *B. fragilis*. Statistical significance was estimated by T-test

Using the taxonomic level information for each sample obtained by QIIME analysis, we constructed stacked graphs of the microbiome proportions for three selected levels, phylum, genus, and species. As shown in Fig. 2a–c, at the phylum level, a majority of the bacterial population (> 75%) in normal tissues consisted of two OTUs, Bacteroidetes and Firmicutes, consistent with what was previously reported [39]. In tumor tissues, these two abundant bacterial OTUs were also highly abundant but in significantly decreased proportions (~ 60%). In contrast, Fusobacterium showed a significantly increased proportion in tumor tissues (~ 23%) compared to that in normal tissues (~ 12%) (Fig. 2b). Actinobacteria was found to be more abundant in normal tissue than in tumor tissues, while Spirochaetes was the opposite, although the proportions of these two bacteria were very low under each condition (Fig. 2a).

Most OTUs were similarly proportionated between normal and tumor tissues at the genus and species levels, but a few bacterial compositions were significantly different between the two tissue conditions (Fig. 2b, c). For instance, at the genus level, *Bacteroides*, *Clostridiales* and *Prevotella* were more abundant in normal tissues; in contrast, *Fusobacterium* and *Treponema* were more enriched in tumor tissues (Fig. 2b). At the species level, *B. vulgatus* and *Faecalibacterium (F.) prausnitzii* were more abundant in normal tissues, whereas *F. nucleatum* and *B. fragilis* were significantly increased in tumor tissue (Fig. 2c). Statistical analysis of differences in bacterial compositions in normal and tumor tissues was performed for some selected bacteria at the phylum and species levels (Fig. 2d, e). Of particular interest are significantly higher amounts of two bacterial species, i.e., *F. nucleatum* and *B. fragilis*, in tumor tissues than in normal tissues because these bacterial species have been repeatedly identified as pathogenic bacteria associated with intestinal inflammatory diseases and even with CRC [13–15, 18, 20]. Additional taxa with significant differences in OTUs between tumor and normal conditions are shown in Additional file 1: Fig. S4.

### Prediction of metabolic pathways exerted by microbiomes in normal and tumor tissues

**Fig. 3:**
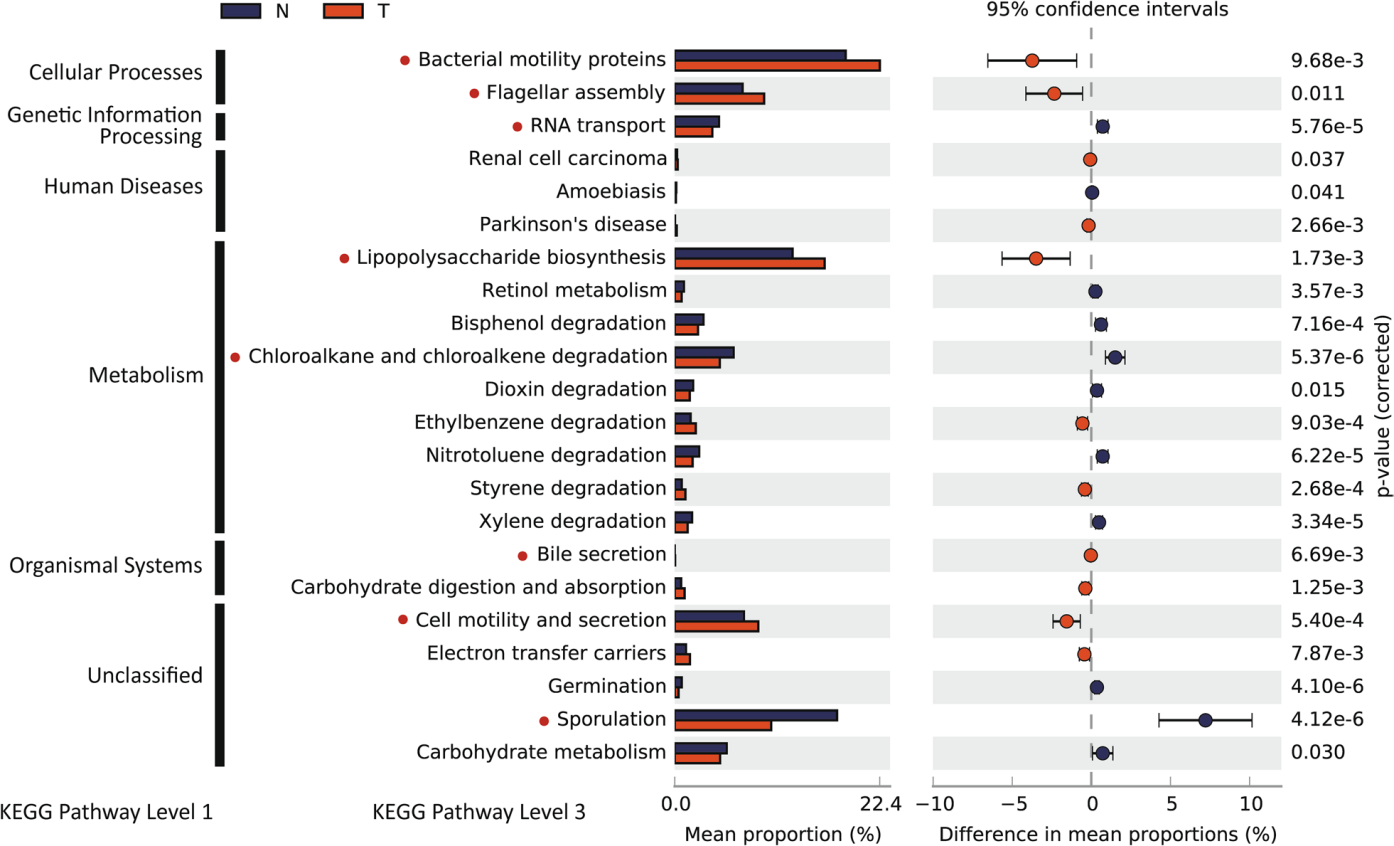
Functional pathways predicted with tumor- and normal tissue-enriched bacteria. KEGG pathways of OTUs enriched differentially between normal and tumor tissues were analyzed using PICRUSt (see “Materials and methods”). P-values were estimated by Welch's t-test

It has been suggested that the cross-talk between the microbiota and the host tissue may be mediated by short-chain fatty acids (SCFAs) produced by the microbiomes. Therefore, to predict microbiome-driven metabolic functions, we used a tool named ‘PICRUSt, i.e., a tool for making inferences by mapping marker genes to known sequenced genomes with information about the identified bacteria and their compositions. In particular, we found that the pathways of production and assembly of bacterial motility proteins (such as flagella) and of lipopolysaccharide biosynthesis were significantly enriched in tumors compared to normal tissues (Fig. 3) (*P* < 0.05), which is consistent with a previous report based on the gut microbiome of Moroccan CRCs [40]. In relation to this observation, it has been reported that overexpression of *flhDC*, a bacterial motility regulator, produced from *Salmonella* is associated with increased tumor cell mass [41]. Another notable compositional difference enriched in tumor tissue was bile acid secretion (Fig. 3) (*P* < 0.01) because it was reported that some of the gut microbiome secretes bile acid, by which the microbiome can provoke a proinflammatory response in hepatic stellate cells [42].

In contrast, the pathways associated with sporulation, RNA transport, and chloroalkane and chloroalkene degradation were more significantly enriched in normal tissues than in tumor tissues (Fig. 3) (*P* < 0.0001), although no good explanation for the cause and effect of this enrichment has yet been provided. Sporulation, i.e., a metabolic event that is expected to occur in gram-positive bacteria such as *Clostridia* [43], which was relatively more enriched in normal tissues, as shown in Fig. 1c, may partially explain this observation.

### Correlation analysis between mRNA expression levels and proportions of bacteria

**Fig. 4:**
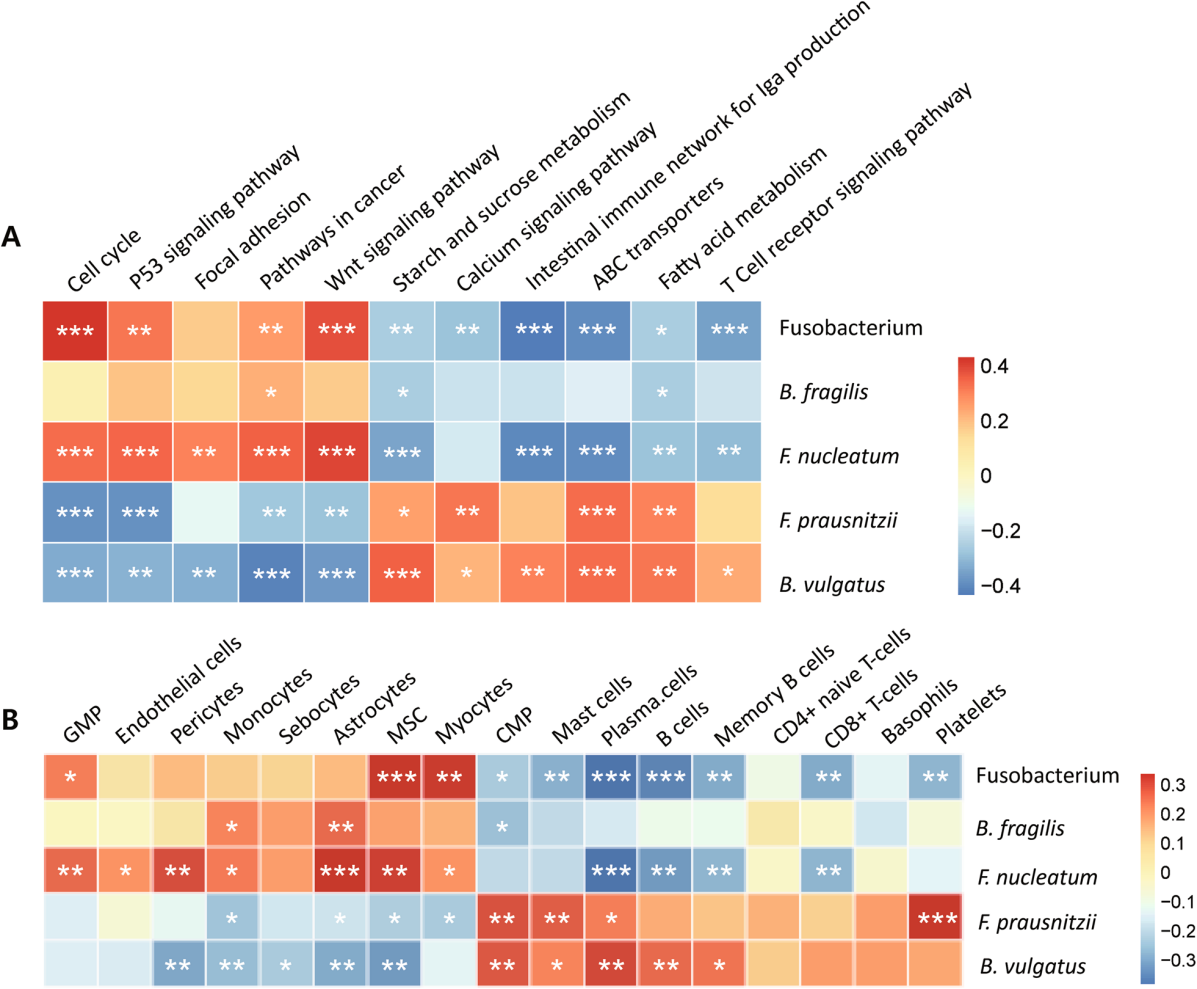
Correlations between the expression levels of host genes and the microbiome composition. **a** The relationship between the abundance of some selected bacteria enriched differently between normal and tumor tissues and the pathways of genes expressed in CRC tissues estimated by ssGSEA.** b** Correlation between the composition of cell types deconvoluted by xCell and the bacteria used in **a**. The color of the squares indicates the magnitude of the correlation according to the scales indicated in the bar on the right side, and asterisks indicate the significance of the correlation (***P < 0.001, **P < 0.01, *P < 0.05)

We then attempted to investigate the functional classes of genes that are differentially expressed in tumor tissues compared to normal tissues, which were conjectured to have been affected by changes in the composition of the microbiota. Briefly, DEGs were estimated by comparing mRNA gene expression in tumor tissues to that in normal tissues, in which functional classes thought to have altered expression together were identified by ssGSEA. Second, a correlation analysis was performed between each of the functional classes in ssGSEA and the proportions of the five bacterial features we selected in Fig. 2 (1 genus; *Fustobacterium*, 4 species; *B. fragilis*, *F. nucleatum*, *F. prausnitzii*, *B. vulgatus*). As a result, we found that the expression levels of host genes had a significant positive or negative correlation with the proportions of tumor-enriched bacteria or normal tissue-enriched bacteria (Fig. 4a), as expected. Interestingly, genes involved in tumor formation, including the cell cycle, cell adhesion, and the *Wnt* signaling pathway, were positively correlated with pathogenic bacteria, including *F. nucleatum* and *B. fragilis*, whereas normal tissue-enriched bacteria including *F. prausnitzii* and *B. vulgatus* were positively correlated with genes involved in starch and sucrose metabolism, the intestinal immune network and ABC transporters.

We also tried to investigate which cell types are likely to interact with microbial communities during tumor formation. After the cell types expected to contribute to bulk RNA-sequencing data were deconvoluted using a program called xCell [44], a correlation analysis was performed in the same way as was done for the DEGs described above. Interestingly, we found that lymphoid cells, including both B and T cells, were positively correlated with normal tissue enriched bacteria, whereas myeloid cells, including monocytes and pericytes were positively associated with tumor-enriched bacteria (Fig. 4b).

### Enrichment of pathogenic bacteria can be a biomarker for better CRC prognosis

**Fig. 5:**
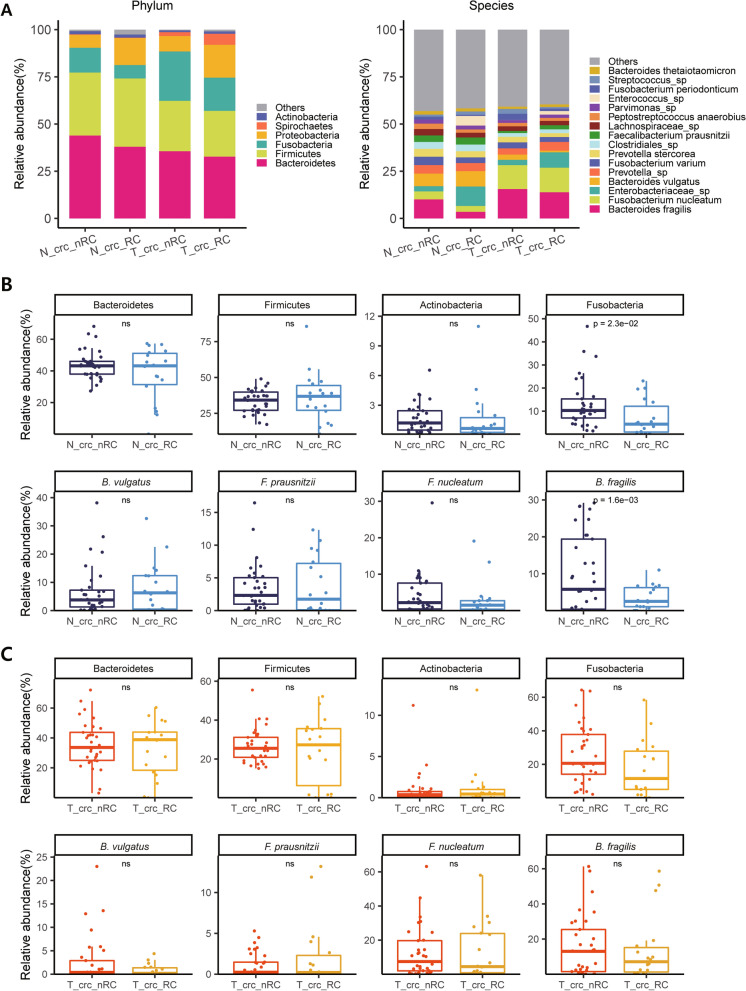
Comparison of the microbiome composition in four different tissue types.** a** Patients were divided into four subgroups by adding prognostic information that was retrospectively determined (RC: recurrence; nRC: nonrecurrence), accompanied by normal tissue- and tumor-derived microbiomes, i.e., ‘N_crc_RC’, ‘N_crc_nRC’, ‘T_crc_RC’, and ‘T_crc_nRC’, as described in the main text. Differences in microbial composition are displayed at the phylum (left) and species levels (right). **b** Box plot analysis of the relative abundance of selected OTUs at the phylum level (top) and the species level (bottom) between ‘N_crc_RC’ and ‘N_crc_nRC’. **c** Box plot analysis of the relative abundance of selected OTUs at the phylum level (top) and the species level (bottom) between ‘T_crc_RC’ and ‘T_crc_nRC’. **a**, **b** The samples used were derived from a total of 33 ‘crc_nRC’ and 18 ‘crc_RC’ samples for both normal and tumor samples (Additional file 1: Table S1)

**Fig. 6:**
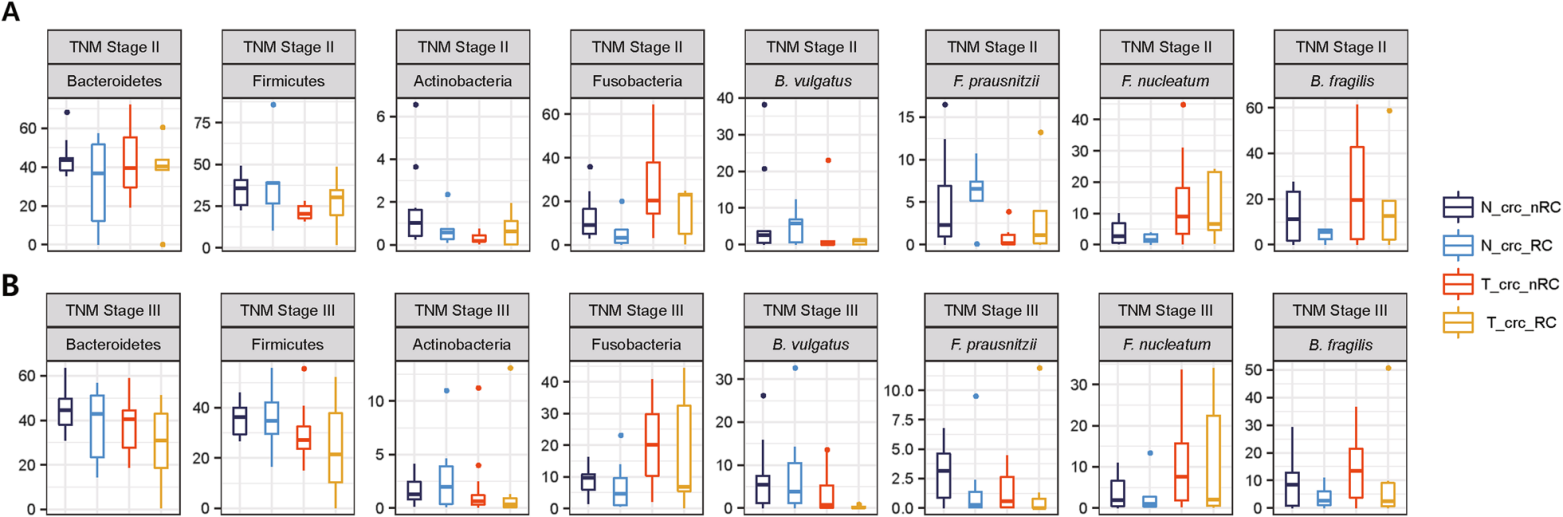
Difference in bacterial abundance between ‘crc_RC’ and ‘crc_nRC’ when the TNM stages were controlled. **a** Comparison of the abundances of selected OTUs for patients with TNM stage II (from 12 ‘crc_nRC’ and 3 ‘crc_RC’ samples) and **b** Comparison of the abundances of selected OTUs for patients with TNM stage III (from 17 ‘crc_nRC’ and 6 ‘crc_RC’ samples). **a**, **b** Refer to Additional file 1: Table S1 for the numbers of samples used

We next wondered whether the microbial composition could predict the prognosis of CRC patients. The proportions of tumor-enriched bacteria and normal tissue-enriched bacteria were compared between the ‘crc_RC’ (patients with recurrence) and ‘crc_nRC’ groups (patients without recurrence). Since normal and tumor tissues were collected from the same individuals, comparisons (‘crc_RC’ *vs.* ‘crc_nRC’) were performed separately for the normal tissue-derived microbiome (i.e., ‘N_crc_RC’ *vs.* ‘N_crc_nRC’) and for the tumor-derived microbiome (i.e., ‘T_crc_RC’ *vs.* ‘T_crc_nRC’). Surprisingly, we found that tumor-enriched bacteria, including *B. fragilis* and *F. nucleatum,* were significantly more abundant in ‘crc_nRC’ than in ‘crc_RC’, indicating that the enrichment of these well-known pathogenic bacteria was associated with better prognosis of CRC patients (Fig. 5), which is contradictory with what has been previously reported [8, 18, 45–47]. To exclude the possibility that these unexpected observations were due to the nonrandom distribution of patients with early or late TNM stages into ‘crc_RC’ and ‘crc_nRC’, the comparison was conducted again after the TNM stages were controlled; comparison of ‘crc_RC’ *vs.* ‘crc_nRC’ was performed only for patients with TNM stage 2 (Fig. 6) and similarly only for patients with TNM stage 3. The samples from TNM stages 1 and 4 were removed because the number of samples was too small (< 9) (Additional file 1: Table S1). Nevertheless, even in the stage-fixed sets, the conclusion was consistent, in that enrichment of pathogenic bacteria was associated with better prognosis of CRC patients.

## Discussion

In the present work, we showed that alterations in the compositions of the microbiome were significantly associated with changes in the host tissue states from normal tissue to tumors, coupled with changes in the levels of some genes expressed in host tissues.

The method of comparing the microbiome compositions obtained under two different physiological conditions is basically similar to many other genomic, transcriptomic, and proteomic data analyses performed in the control-case design. However, finding biologically meaningful associations between the composition of the microbiome and human disease is not easy for several reasons. First, microbiomes are highly heterogeneous, to the extent to which even the same individual can carry varied microbiomes depending on diets or physiological states, not to mention that different individuals have different microbial compositions in the same tissue type. Second, various sample sources, such as fecal samples, mucus samples or tissue samples of patients, are used to isolate the microbiome. Third, various sequencing methodologies, either whole genome shotgun sequencing or 16S rRNA amplicon sequencing methods, are chosen to generate source sequencing data to identify microbiome components. Therefore, conclusions are often inconsistent regarding the increase or decrease in certain bacterial species associated with a given human disease, and the microbiome related to CRC is no exception. However, two bacterial species, *F. nucleatum* and *B. fragilis*, are consistently reported to increase in feces or mucus from CRC patients compared to healthy individuals or at increased levels in tumor tissue compared to normal tissue of the same CRC patient [8, 48, 49]. It seems that *F. nucleatum* is the most studied bacterial species related to the onset or progression of CRC. Our present study also drew the same conclusion. Taken together, the two bacteria, *F. nucleatum* and *B. fragilis*, may have a causal relationship in provoking inflammatory diseases and cancers.

As expected, these pathogenic bacteria have been reported to be associated with poor prognosis in CRC patients. For instance, patients with a high amount of *F. nucleatum* tended to have shorter survival times than patients with a low amount of *F. nucleatum* [45–47]. Yu et al. [50] showed that *F. nucleatum* was more enriched in chemoresistant recurrent CRC patients than in chemosensitive nonrecurrent patients by triggering the autophagy pathway via the TLR4/MYD88 pathway, which is consistent with the results of Zhang et al. [51]. However, we observed otherwise in the present work, showing that these two pathogenic bacteria were more enriched in CRC patients without recurrence (i.e., ‘crc_nRC’) than in CRC patients with recurrence (i.e., ‘crc_RC’). As shown in Figs. 5 and 6, patients with higher levels of pathogenic bacteria in their tissues had a consistently better prognosis, regardless of the sources of microbiomes (i.e., tumor- or normal tissue-derived microbiomes).

Interestingly, some studies have reported a good prognostic association of *F. nucleatum* in CRC. For instance, according to Oh et al. [52], the survival of *F. nucleatum*-high CRC patients was better than that of *F. nucleatum*-low CRC patients, when only a subgroup of microsatellite-stable CRC patients with nonsigmoid colon cancers treated with oxaliplatin-based chemotherapy were separately investigated. Notably, both Oh et al.’s samples and ours are based on microbiome data generated by the 16S rRNA amplicon sequencing method for tissue samples of homogenous Korean-only CRC patients. Saito et al. [53] showed that *F. nucleatum* could be associated with a good prognosis in a subgroup of CRC patients with *FOXP3*^*lo*^ non-Treg cell infiltration.

Unfortunately, no good explanation has yet been proposed for this unexpected link between pathogenic bacteria and a good prognosis, unlike the case for the association with a poor prognosis. It is possible that there are strain-to-strain differences in the bacterial species present in different ethnic populations or that differences in the genetic makeup or local diet can cause the same pathogenic bacteria to have a different effect in the individuals tested. In addition, another possibility can be clued from the relationship between the density of *F. nucleatum* and the density of tumor-infiltrating lymphocytes (TILs); the density of *F. nucleatum* was reported to be positively correlated with the density of TILs in some CRCs [54], and the high density of TILs was shown to be associated with a better prognosis in CRC [55]. Therefore, it will be a great opportunity to develop a microbiome-based prognostic marker in the future if we can determine how these pathogenic bacteria can inhibit the recurrence of cancer after surgical treatment and chemotherapy.

## Conclusions

We investigated whether alterations in the compositions of the microbiome were significantly associated with changes in the host tissue states from normal tissue to tumors, coupled with changes in the levels of some genes expressed in host tissues. We showed that the two pathogenic bacteria, *F. nucleatum* and *B. fragilis*, that were more abundant in tumor tissues than normal tissues were surprisingly more abundant in the patients without recurrence than in the patients with recurrence. We believe that our study will contribute to exploring the composition of tissue microbiomes that is critical in predicting the prognosis of CRC patients.

## Supplementary Information

**Additional file 1.** Additional figures and table.

**Additional file 2.** Taxa at species level.

## Data Availability

The raw data is publicly available on NCBI portal at Sequence Read Archive (SRA) BioProject ID: PRJNA743150. 16S rRNA microbiome data—Submission ID: SUB9930275 and RNA-Seq data—Submission ID: SUB9954281.
