# Supplementary material for: Analysis of changes in microbiome compositions related to the prognosis of colorectal cancer patients based on tissue-derived 16S rRNA sequences
Source: J Transl Med. 2021 Nov 29;19:485. doi: 10.1186/s12967-021-03154-0 (PMC8628381; doi:10.1186/s12967-021-03154-0)
Supplement: Supplementary file 1 — Additional file 1. Additional figures and table. [file 12967_2021_3154_MOESM1_ESM.docx]

**Analysis of changes in microbiome compositions related to the prognosis of colorectal cancer patients based on tissue-derived 16S rRNA sequences**

Sukjung Choi^1^, Jongsuk Chung^2^, Mi-Ra Jo^3^, Donghyun Park^2^, and Sun Shim Choi^1*^

^1^Division of Biomedical Convergence, College of Biomedical Science, Institute of Bioscience & Biotechnology, Kangwon National University, Chuncheon 24341, Korea

^2^GENINUS Inc., Seoul 05836, Korea

^3^Department of Medical Life Science, College of Medicine, Catholic University of Korea, Seoul 06591, Republic of Korea

*Corresponding author:

Tel: +82-33-250-6487

E-mail: schoi@kangwon.ac.kr

**Additional Table and Figures:**

**Table S1.** Clinical characteristics of the enrolled participants

| **Characteristics** | **crc_nRC (n=33)** | **crc_RC (n=18)** | ***P* value** |
| --- | --- | --- | --- |
| Age (mean±SD) | 62.06±11.06 | 64.83±11.26 | 0.230^*^ |
| BMI (mean±SD) | 23.85±2.85 | 23.12±3.06 | 0.241^*^ |
| **Tumor Locations** |  |  |  |
| Ascending colon | 7 (21.2%) | 3 (16.7%) | 1^*^ |
| Transverse colon | 1 (3%) | 1 (5.6%) | 1^*^ |
| Descending colon | 14 (42.4%) | 7 (38.9%) | 1^*^ |
| Rectum | 10 (30.3%) | 6 (33.3%) | 1^*^ |
| Cecum | 1 (3%) | 1 (5.6%) | 1^*^ |
| tumor size (mean±SD) | 6.13±1.66 | 6.04±2.19 | 0.836^*^ |
| **TNM Stage** |  |  |  |
| Stage 0 | 1 (3%) | - | 1^#^ |
| Stage I | 2 (6.1%) | 1 (5.6%) | 1^#^ |
| Stage II | 12 (36.4%) | 3 (16.7%) | 0.422^#^ |
| Stage III | 17 (51.5%) | 6 (33.3%) | 0.607^#^ |
| Stage IV | 1 (3%) | 8 (44.4%) | 0.009^#^ |

*Unpaired two sample t-test

#Pearson's Chi-squared test


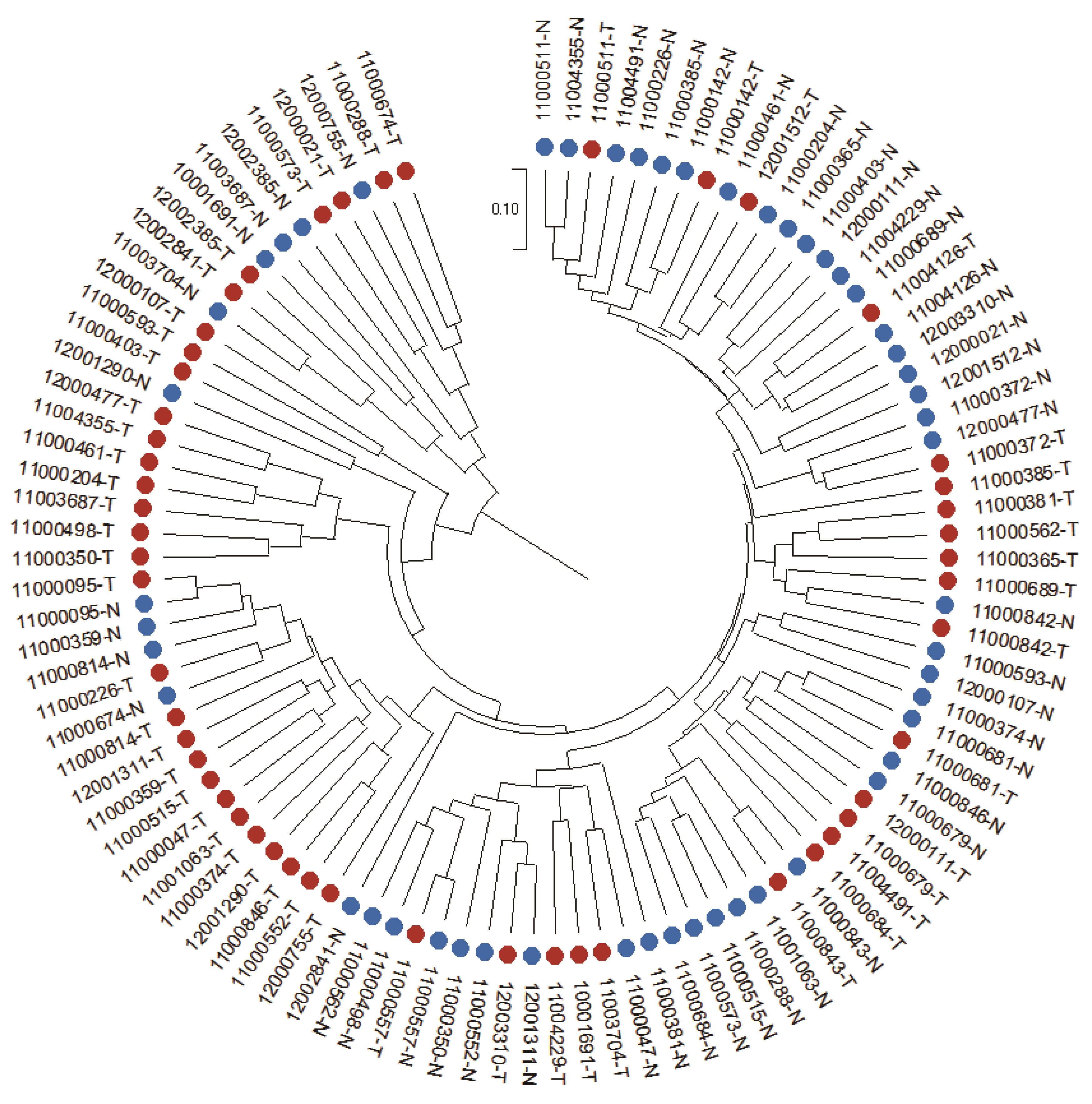


**Fig. S1** Hierarchical clustering of ‘phylotypes’ based on OTUs derived from the CRC tissues of 51 Korean patients. A dendrogram was constructed by the UPGMA weighted UniFrac method.


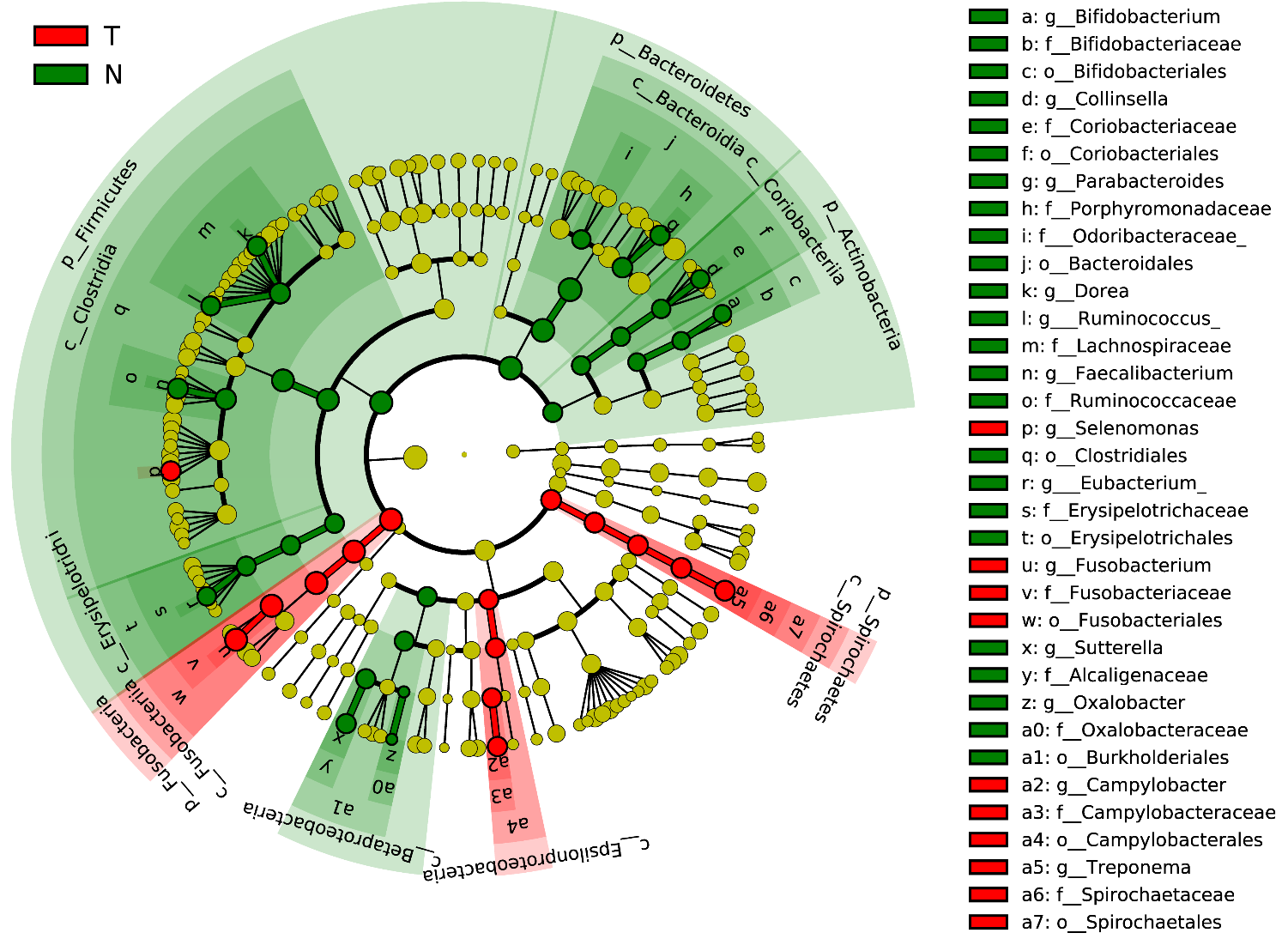


**Fig. S2** Differential enrichment of bacteria in normal and tumor colon tissues. A cladogram was produced by LEfSe: (red) taxa enriched in tumor tissues, (green) taxa enriched in normal tissues.


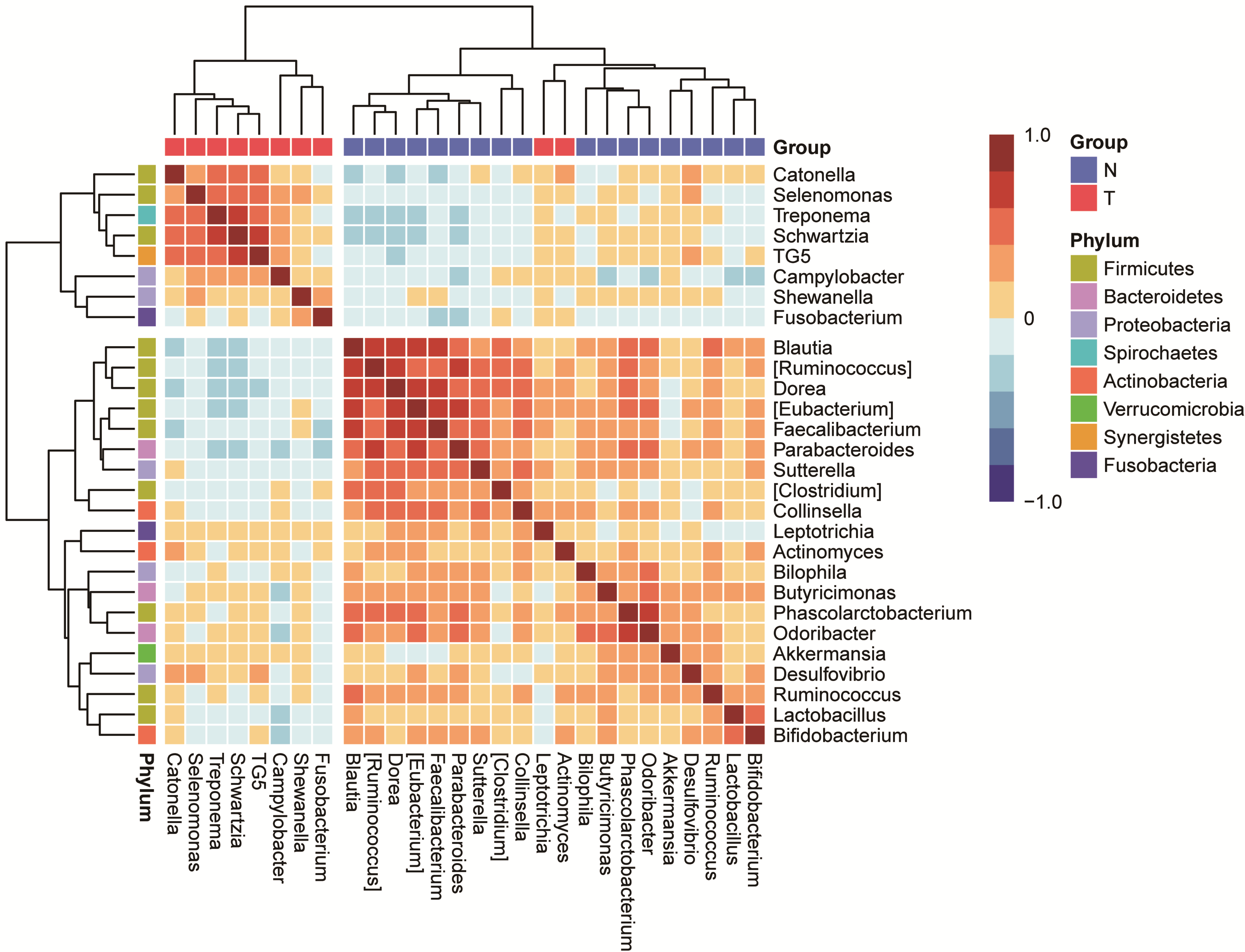


**Fig. S3** The genera co-occurring in normal and tumor tissues. Heatmap of Spearman correlation coefficients of 28 genera in normal (18) and tumor (10) tissues.


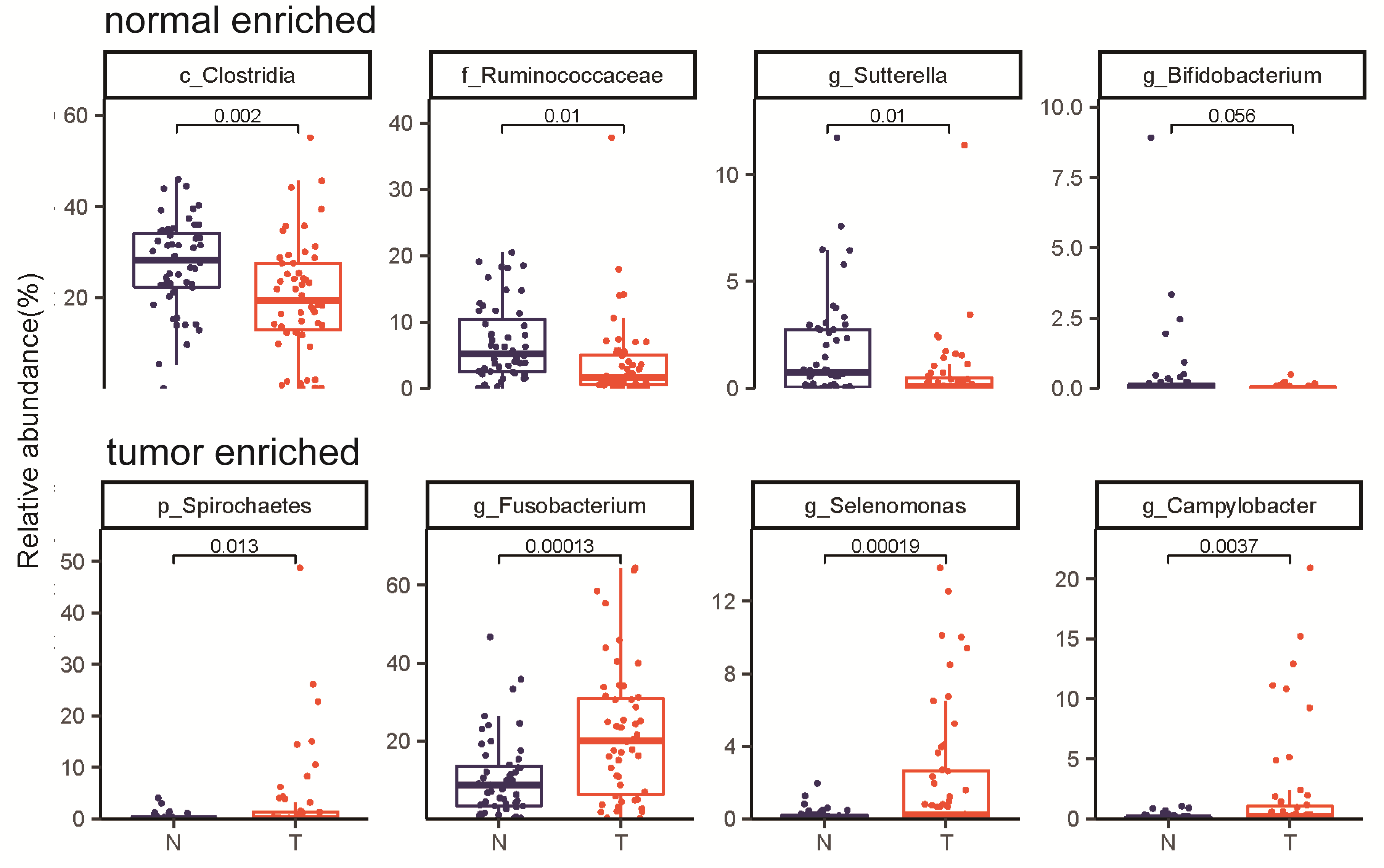


**Fig. S4** Additional taxa showing differences in composition between tumors and normal tissues. N: normal, T: tumor
